# The wound healing and hypoglycemic activates of date palm (*Phoenix dactylifera*) leaf extract and saponins in diabetic and normal rats

**DOI:** 10.1371/journal.pone.0308879

**Published:** 2024-09-23

**Authors:** Hanan S. Anbar, Naglaa Gamil Shehab, Ayah Yasin, Lana Mazen Shaar, Ruba Ashraf, Zahraa Rahi, Raneem Alamir, Deema Alsabbagh, Aya Thabet, Israa Altaas, Yosra A. Lozon, Nadia M. M. El Rouby, Aliasgar Shahiwala

**Affiliations:** 1 Department of Pharmaceutical Sciences, Dubai Pharmacy College for Girls, Dubai, United Arab Emirates; 2 Pharmacognosy Department, Faculty of Pharmacy, Cairo University, Cairo, Egypt; 3 Undergraduate Student, Dubai Pharmacy College for Girls, Dubai, United Arab Emirates; 4 Department of Biomedical Sciences, Dubai Medical College for Girls, Dubai, United Arab Emirates; Alexandria University, EGYPT

## Abstract

**Introduction:**

Indigenous plants have historically been crucial in treating human diseases across various cultures worldwide. Research continues to uncover new therapeutic uses for indigenous plants, from treating infectious diseases to managing chronic conditions such as diabetes and wound care. This study aimed to examine the effect of palm tree leaves "*Phoenix dactylifera L*" extract and its topical film formulation on wound healing and blood glucose levels.

**Methods:**

Palm leaves were collected, authenticated, powdered, and extracted with ethanol by cold maceration. Saponins were isolated. The dried extract was analyzed using reverse-phase high-pressure liquid chromatography to identify the phytochemicals present. Diabetes mellitus was induced by a single intraperitoneal injection of Streptozotocin (40mg/kg). Rats with blood glucose levels ≥ 200 mg/dl were used to determine the reduction in blood glucose with or without the oral extract. Incision and excision wounds were induced in both diabetic and normal rats. Topical films containing extract or saponin and inert films were applied to the wounds every other day, and wound sizes were recorded until the wound was completely healed.

**Results:**

The presence of six flavonoids, Naringin, Rutin, Quercetin, Kaempferol, Apigenin, and Catechin, and five phenolic acids, Syringic acid, p Coumaric acid, Caffeic acid, Ferulic acid, Ellagic acid were detected in the dried extract. A significant reduction in blood sugar in diabetic rats and wound diameter in the treated group compared to the control group in both diabetic and normal rats was observed, confirming the promising role of palm leaf extract on diabetes and wound care. Macroscopic, morphometric, and histological data suggested that the cutaneous wound healing in rats treated with the leaf extract was better and faster than the control or inert groups.

**Conclusions:**

Our research findings highlight the marked effect of *Phoenix dactylifera* extract as a supportive or alternative treatment for both hyperglycemia and incision or excision wounds. Further research and clinical trials are warranted to validate these findings and explore the underlying mechanisms of action.

## Introduction

Diabetes mellitus is a group of metabolic disorders that cause hyperglycemia because of impaired insulin synthesis and activity. Hyperglycemia increases a patient’s risk of comorbid diseases that affect multiple organ systems. Furthermore, diabetes impairs wound healing as one of its main consequences [1]. Diabetic ulcers are one of the most severe problems across the world and every year, millions of diabetic individuals’ lower limbs are amputated due to wound progression [2]. Diabetic ulcers are more complicated to develop and grow than other chronic wounds due to the involvement of multiple pathological causes such as neuropathy, foot deformities, vascular disorders, and infections [3].

Wound healing has been divided into three stages: inflammation, proliferation, and remodeling. After the injury, the inflammation starts with vasoconstriction, and the release of inflammation is caused mainly by fibroblasts and the angiogenesis process. The remodeling stage is distinguished by reformulations and improvements in collagen fiber components that boost tensile strength [4, 5]. An imbalance in the formation of free radicals and antioxidants has been reported to cause oxidative stress, tissue damage, and delayed wound healing. As a result, eliminating reactive oxygen species (ROS) could be an essential technique in the healing of chronic wounds [6]. Diabetes can affect wound healing and make it more challenging for the body to repair damaged tissues. People with diabetes often experience slower wound healing and are at a higher risk of developing complications associated with wounds.

Many medications are used to manage elevated blood sugar, but herbal alternatives are always preferable [7]. It is common knowledge and practice in Asian and African nations to employ medicinal herbs to cure wounds, boils, ulcers, and sores [7–10]. According to a clinical study report, herbal formulations in patients with diabetic foot ulcers prevented 85% of legs from being amputated, proving the effectiveness and consistency of medicinal plants [11].

In ancient cultures and throughout major civilizations, including the Biblical tradition, dates (*Phoenix dactylifera*) have been regarded as preventive or curative foods [12]. In folk medicine in America and Africa, palms are often used to treat infections and disorders in the digestive, respiratory, cardiovascular, dermal, endocrine, genitourinary, muscular, skeletal, neural, and mental systems [13]. Many countries, including the United States and southwest Iran, use palm to reduce diabetes-related blood glucose levels [14–16]. Epicarp, a flavonoid from date fruits, significantly improved various biochemical outcomes in diabetic rats, according to a recent study on flavonoid components from date fruits [17]. An earlier study found that the leaves had antioxidant, anti-inflammatory, and anti-tumour properties that have therapeutic effects in treating various illnesses. This gives rise to optimism for new treatment approaches [18].

Therefore, this study was undertaken to extract bioactive compounds from palm leaves, prepare topical and oral dosage forms from the total extract and isolated saponins, evaluate the potential of the prepared dosage forms for wound healing in both normal and diabetic rats, and study the possible antidiabetic activity of the plant extract.

## Materials and methods

### Preparation of plant extract

The leaves of *Phoenix dactylifera* were collected from the Muhaisnah area of Dubai on 20 August 2022. The trees belonged to the college campus, and the management gave the necessary permission. Dr. Naglaa Shehab, Department of Pharmaceutical Sciences, Dubai pharmacy college for girls identified and authenticated the plant. Voucher samples were kept (1-9-22#) in the pharmaceutical sciences department of the Dubai Pharmacy College for Girls. The leaves were air-dried and powdered (the leaves weighed 1052.4 g, and the powder was 686.2 g). The powdered leaves were extracted by cold maceration (for one week) with 50% alcohol (2 liters). The extract was filtered and evaporated using a rotary evaporator at 50°C under reduced pressure, while the remaining solvents were removed using a lyophilizer (BK FD10P, Biobase, China). The part of the dried extract was kept for isolation of the saponin and further formulation and biological studies.

### Isolation of saponin content

The portion of the extract (50 g) was dissolved in 20 ml of ethanol (96% v/v). Acetone was added to the solution drop by drop until complete precipitation appeared. The precipitate was separated by decantation and washed with acetone. Finally, the saponins (8 g) were dried under reduced pressure using a rotary evaporator at 40°C [19].

### Standardization of plant extract

#### Colorimetric determination of phenolic and flavonoid contents

The extract’s total flavonoid and phenolic contents were determined spectrophotometrically (UV1800, Shimadzu, Japan). The total phenolic content was estimated using the Folin Ciocalteu method [20]. The results were represented as g/100g gallic acid equivalents based on the dry weight of the plant material, using the gallic acid as a standard (λ_max_ 750 nm). Similarly, total flavonoids were determined by the aluminium chloride method using quercetin as a standard [21].

All experiments were carried out in triplicate.

#### Reverse Phase (RP) HPLC analysis of the *Phoenix dactylifera* extract

The composition of *Phoenix dactylifera* extract was evaluated using RP HPLC analysis by an Agilent 1100 HPLC system equipped with a C18 MS packed column (5 μm, 4.6 mm i.d. × 125 mm). The 10 μL extract or standards were injected into the column. Separation of phenolic acid compounds involved employing a gradient mobile phase of methanol and acetic acid in water (1:25 v/v) at λ_max_ 280 nm [22]. Identification of flavonoids was performed at λ_max_ 360 nm using 1ml/min isocratic flow of a binary mixture of methanol /water (50:50 *v/v*), adjusted to pH 2.8 with phosphoric acid [23] The picks were compared against the retention times of the reference standards.

#### Preparation and evaluation of film formulations

The film formulation was prepared for topical application for wound healing as per the method reported earlier [24].

### Film containing plant extract

A 400 mg of alcohol extract, 600 mg polyvinyl alcohol (PVA) and 50 mg tween 80 were dissolved in a 10 ml mixture of methanol and water (1:1) (Table 1).

**Table 1:** Optimized film composition.

Ingredient	Film containing Plant Extract	Film containing Saponins
Plant Extract	400 mg	--
Saponins	--	400 mg
Polyvinyl Alcohol	600 mg	600 mg
Tween 80	50 mg	75 mg
Methanol: water (1:1 v/v)*	10 mL	--
Water*	--	10 mL

* removed during drying

The solution obtained was transferred to a Petri dish and subjected to initial drying on a hot plate at 60°C, followed by further drying overnight in a hot air oven. Once completely dried, the films were meticulously removed from the Petri dish and sliced into strips measuring 2x2 cm for additional analysis.

### Film containing saponin

400 mg of saponin, 600 mg PVA, and 75 mg tween 80 were dissolved in 10 ml water (Table 1). The resulting solution was transferred to a Petri dish and was dried at 60°C in a hot air oven overnight. After drying, films were carefully removed from the Petri dish and cut into 2x2 cm strips for further characterization.

### Inert film

600 mg PVA and 75 mg tween 80 were dissolved in 10 ml water. The resulting dispersion was transferred to a Petri dish and dried at 60°C in a hot air oven overnight. After drying, films were carefully removed from the Petri dish and cut into 2x2 cm strips for further characterization.

### Weight variation

Three randomly selected films measuring 2x2 cm^2^ were obtained from each batch of film formulation to assess weight variation. Each film was individually weighed using an electronic balance, and the average weight for each batch was calculated.

### Film thickness

The thickness of the films was measured at five different positions, including the center and the four corners. Digital Vernier calipers were used for this measurement, and the process was repeated three times. The average thickness along with the standard deviation was calculated.

### pH

The pH of the film was determined by dissolving it in 100 ml of double distilled water. A digital pH meter was used to measure the pH of the resulting solution.

### Drug content determination

A stock solution of the extract was prepared by dissolving around 20 mg of extract in methanol to obtain a concentration of 200 μg/ml. From this stock solution, various samples were prepared by taking 1 ml, 2 ml, 3 ml, and 4 ml and diluting them to 5 ml with methanol. These samples contained extract concentrations of 40 μg/ml, 80 μg/ml, 120 μg/ml, and 160 μg/ml, respectively. The absorption maxima (λmax) were determined by scanning the 120 μg/ml solution against the reagent blank using a UV visible spectrophotometer. Four λmax values were obtained: 412 nm, 435 nm, 470 nm, and 665 nm. The absorption of the prepared solutions was measured at the 470 nm absorption maxima against the reagent blank, as it provided the highest R^2^ value and no interference from chlorophyl at this λmax. The readings were recorded in triplicate, and the absorption mean values (n = 3) were plotted graphically against the concentration.

To assess the uniformity of drug content and determine the drug assay, an accurately weighed portion of the film, approximately 100 mg, was completely dissolved in 10 mL of methanol. The drug content was then determined by utilizing a calibration curve after appropriate dilution of the sample. This test was repeated for three different films.

### Moisture content

The prepared films were individually weighed and placed in a desiccator containing calcium chloride at room temperature for 24 hours for moisture content analysis. The films were reweighed at specified intervals until a constant weight was achieved. The percentage of moisture content was calculated using the following formula:

$$\%\;Moisture\;Content=\frac{(Initial\;Weight\;-\;Final\;Weight)x100\;}{Final\;Weight\;}$$

### Folding endurance

The number of times film was folded at the same location until it broke was considered folding endurance.

### Tensile strength

Tensile strength was determined by applying the load on the film till it broke. The applied load at the point of rupture was divided by the cross-sectional area of the strip to determine the tensile strength, as indicated by the following equation:

$$Tensile\;strength=\frac{Force\;at\;breakage\;(kg)}{Film\;thickness\;(mm)xfilm\;width\;(mm)}$$

### Percent elongation

Percent elongation refers to the extent of stretching experienced by a film sample (2 × 2 cm^2^) when subjected to stress. Strain, which represents the deformation of the strip before it breaks under stress, is the basis for calculating percent elongation. The percent elongation is determined using the following formula:

$$\%\;Elongation=\frac{Increased\;in\;length\;of\;strip\;(mm)x100}{Initial\;length\;of\;strip\;(mm)}$$

### Contact angle and wetting time

To measure the contact angle, a drop of double-distilled water is placed on the surface of the dry film. A digital camera captures images of the water droplet within 10 seconds of deposition. The digital pictures are analyzed using ImageJ 1.28v (NIH, USA) software to determine the angle. The time taken for the film to completely absorb the drop is considered the wetting time.

### Animal studies

#### Animals

Thirty-five female Wister rats weighing 230±20 g was used in this study. The rats were kept in a controlled environment with a temperature of 25°C and a light/dark cycle of 12 hours with free access to food along with water. All animal studies were carried out in accordance with ethical norms and with the agreement of the Dubai Pharmacy College for Girls’ Research and Ethics Committee in Dubai, UAE (ethical approval #REC/UG/2022/03).

#### Change in blood glucose level

Group 1, consisting of five normal rats, received a single I.P. saline injection followed by oral 5% CMC. In the remaining animals, DM was induced by a single intraperitoneal injection of Streptozotocin 45 mg/kg [25]. Two days later, the fasting blood glucose level was measured using a glucometer (standard glucose monitor). Animals with more than 200 mg/dl of blood glucose were considered diabetic and used in this study. Fifteen diabetic rats were divided into three groups of five rats each as follows:

Group 2: treated with a single oral dose of saponin (40 mg/kg/day in 0.5% CMC); Group 3: treated with an oral dose of palm leaves extract (100 mg/kg/day in 0.5% CMC, orally); Group 4: treated with an oral dose of metformin as a standard drug (100 mg/kg/day in 0.5% CMC) (Table 2). The fasting blood glucose levels were recorded before the treatment and 2, 4, 6, and 24 hours after the treatment.

**Table 2:** Treatment groups in blood glucose level test.

Group	Treatment
Group 1 (Control group)	A single I.P. saline injection followed by oral 0.5% CMC
Group 2 (Dia-Saponin group)	An oral dose of saponin (40 mg/kg/day in 0.5% CMC)
Group 3 (Dia-Extract group)	An oral dose of palm leaves extract (100 mg/kg/day in 0.5% CMC, orally
Group 4 (Dia-Metformin group)	An oral dose of metformin as a standard drug (100 mg/kg/day in 0.5% CMC)

### Wound healing study

**Fig 1:**
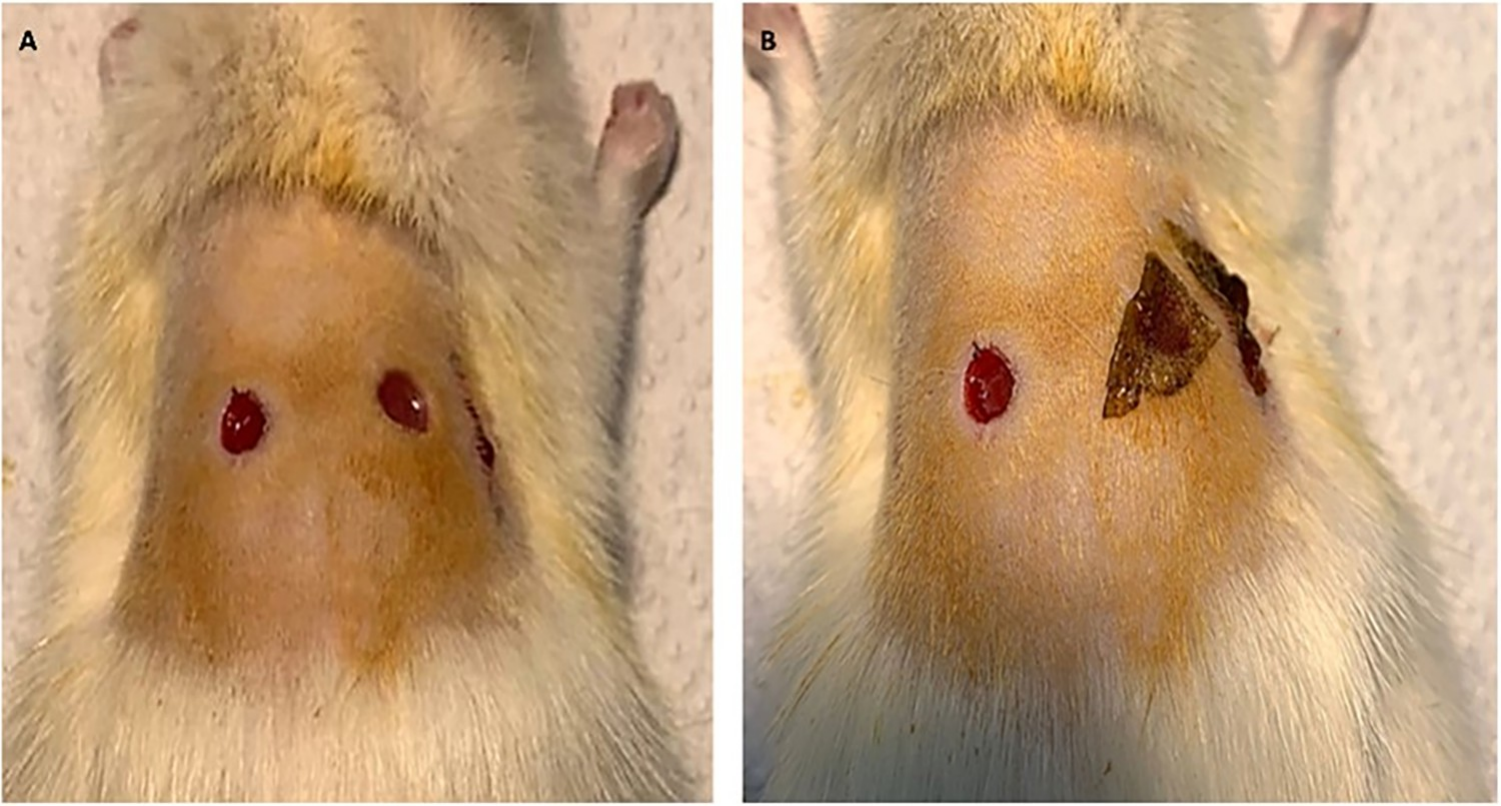
**A**) Excision and incision wound model without treatment, **B**) the wound after application of topical film.

The wound healing effect was studied in two models, the normal rat model and the diabetic rat model, in which the wound was induced in normal or diabetic rats, and the effects of the extract, saponin, and inert films were studied as follows:

To study the wound healing effect, normal or diabetic rats were first anesthetized by intraperitoneal injection of a mixture of Ketamine and Xylazine 0.1 ml/kg which act through various mechanisms to induce anaesthesia and providing potent analgesic effect. Therefore, it can alleviate the suffering and pain to animals [26]. The dorsal skin hair of the anesthetized rats was shaved bilaterally using an electric razor, and the shaved area was sterilized using a povidone-iodine 10% solution. Two circular full-thickness skin excision wounds were made using a biopsy punch of 5 mm diameter. Two longitudinal, 1.5 cm incision wounds were made using a sterilized scalpel. The wounds were left open and not sutured, as shown in Fig 1. The rats were transferred to a warm place and monitored for recovery from anaesthesia. Animals were kept separated in individual sterilized cages with full access to water and food. Rats were closely monitored for any signs of infection and excluded from the research if infected. Assuming the day on which wounds were created as day 0, extracts and saponin were administered topically as a film to the wounds, and wound size was assessed every two days [27].

After the creation of the excision wounds in both normal and diabetic rats, they were randomly divided in to three groups receiving treatment with inert film, extract film and saponin film respectively [28]. Simultaneously incision wounds were made diabetic rats and treated with inert or extract film respectively. Topical films containing extract or saponin and inert films were applied to the wounds every other day, and wound sizes were recorded until the wound was completely healed. The wound size and diameter were measured with a digital Vernier Calliper every two days until each wound was fully closed. The repeated measures ANOVA (IBM SPSS Statistics, version 27) was used to compare the differences in the wound diameter and differences are considered significant at p < .05.

Following the complete closure of the wound, the animals were sacrificed at the end of the experiment by an overdose of anaesthesia followed by cervical dislocation. The skin fragments were collected for histopathological investigations. The obtained skin specimens were fixed in 10% formalin saline for a day. Paraffin blocks were processed, and 5 μm thick sections were cut and stained with Haematoxylin and eosin (H&E) to identify the structural changes and Mallory’s trichrome staining to identify collagen fibre deposition.

## Results and discussion

### Standardization of the plant extract

**Fig 2:**
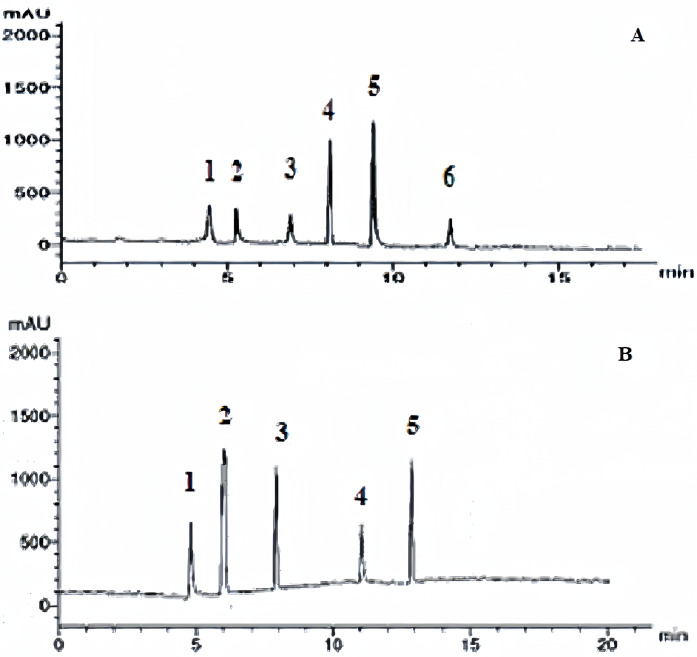
Identification of the flavonoid constituents by RE HPLC chromatogram. **A**. flavonoids, **B**. Phenolic acids.

Colorimetric determination of phenolic and flavonoid contents revealed that the extract of the leaves has 0.4g/100 g as phenolic content and 4.56% as flavonoid content. RP HPLC evaluation of the leaf extracts enabled the identification and quantitation of various flavonoid and phenolic compounds, as shown in Tables 3 and 4 and Fig 2A and 2B. Six flavonoids and five phenolic acid compounds were detected in the extract of the leaves. The total percentages of the flavonoid and phenolic acid compounds were 3.545% and 4.838% respectively. Apigenin and Kaempferol were the major flavonoid components 1.467% and 1.287%, respectively) while Caffeic acid, p Coumaric acid, Ellagic acid (1.98, 1.123, and 0.875, respectively) were the major phenolic acid components in the leaves extract.

**Table 3:** Identification of the flavonoid constituents by RE HPLC.

No.	Rf value	Constituent	Percentage
**1**	4.4	Naringin	0.233
**2**	5.2	Rutin	0.278
**3**	7.0	Quercetin	0.127
**4**	8.0	Kaempferol	1.287
**5**	9.8	Apigenin	1.467
**6**	12.0	Catechin	0.153
	Total	6	3.545

**Table 4:** Identification of the phenolic acid constituents by RE HPLC.

No.	Rf value	Constituent	Percentage
**1**	5.0	Syringic acid	0.514
**2**	6.0	p Coumaric acid	1.123
**3**	8.0	Caffeic acid	1.98
**4**	11.0	Ferulic acid	0.346
**5**	13.0	Ellagic acid	0.875
	Total	5	4.838

### Film formulation and characterizations

The local administration of the drug on the skin is used for topical treatment of skin diseases as well as systemic delivery through transdermal absorption. Commonly used topical dosage forms are semisolids, such as ointments and creams, which have a sticky and greasy feel after application, leading to poor patient compliance [29]. Also, they can be easily wiped off by the patient’s clothes, and hence, repeated application is required in case of chronic wound healing. Adhesive bandage has various drawbacks like skin irritation, occlusive properties that do not allow air and water vapor to pass through, difficulty in applying on curved surfaces, pain while peeling off, and poor aesthetic appeal [30]. To help facilitate wound healing, wound dressings should cover the wound, maintain the body’s water and air permeability, and prevent infections [31, 32]. Therefore, there is a need for the development of a dosage form that permits less frequent dosing by maintaining close contact with the skin for a prolonged period, thereby improving patient adherence [31]. The film formulation presents a simple system for both topical and transdermal drug delivery through the skin. Among its advantages are transparency, a non-greasy texture, reduced skin irritation, wipe-off resistance, longer retention, increased dosage flexibility, and improved patient compliance. [33].

Polyvinyl alcohol (PVA) is a neutral hydrogel widely used for controlled-release wound healing applications, exhibiting high biocompatibility, hydrophilic properties, and biomechanical properties [34, 35] Considering these properties, PVA was selected as a film-forming polymer. Tween was added to improve the flexibility and wettability of the film. The prepared film was found to have optimum physical, mechanical, and wetting properties and a neutral pH to avoid any irritancy to the skin. The applied films stayed on the wound for 24 hrs, ensuring a slow release of active constituents over a period.

The calibration curve of the extract was developed in the methanol to determine the extract content uniformity of drug content and determine the drug assay in the film. The calibration curve suggested the linearity and consistency with Beer Lambert’s law in the 40–200 μg/ml range at λmax 470 nm.

As depicted in Table 5, the optimized films were evaluated for various physicochemical properties. The film was a non-sticky, smooth texture with high mechanical strength and flexibility. The neutral pH of the film was appropriate for skin applications. Also, the extract was within the 95% to 105% range, indicating a uniform distribution across the film. The folding endurance of the films determines film flexibility and ability to withstand repeated folding under extreme conditions. The folding endurance value directly correlates with the mechanical strength of the film [36]. Tensile strength is the maximum stress applied to a specific point on a strip specimen before it breaks. Generally, the strip’s elongation increases with higher plasticizer content. The contact angle is an important property that provides insights into the film’s wetting behaviour and contact with the wound.

**Table 5:** Evaluation of films.

Evaluation Test	Results (Mean ± SD)
Average weight	167mg ± 4mg
Tackiness	No tacky
Folding Endurance	>100
Thickness	0.38mm ± 0.06 mm
Weight variation [2x2 strips]	245 mg ± 12mg
Tensile strength	0.6 MPa
%Elongation	14.29%
pH	7.1 ± 0.2
Extract assay and content uniformity	97.8±2.3%
Moisture content	2.8±0.9%
Contact angle	76 ± 5°
Wetting time	45 ± 2.1 sec

### Change in blood glucose level

**Fig 3:**
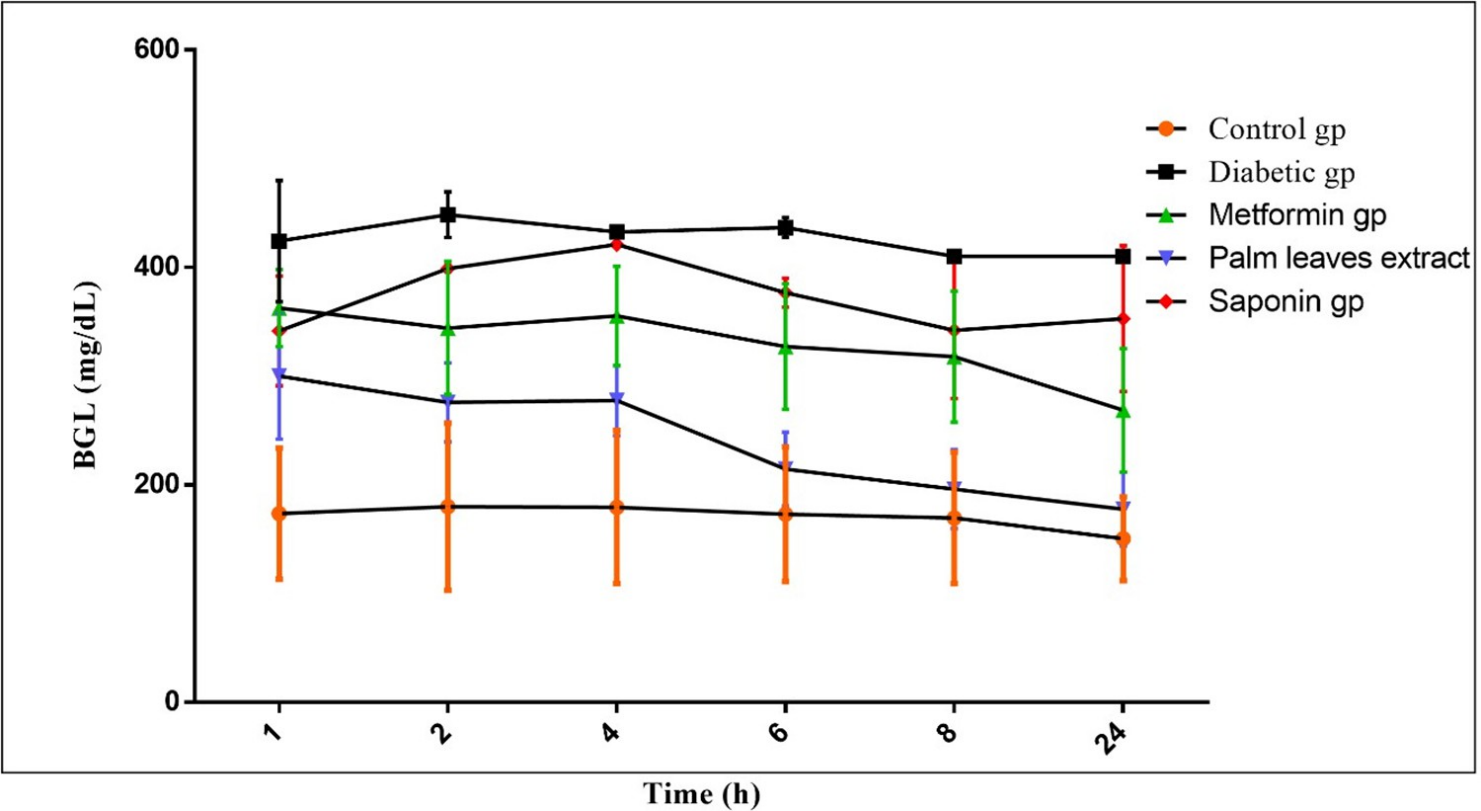
The differences in blood glucose levels after oral treatments with alcoholic extract, saponin, and metformin over 24 hours.

Fig 3 demonstrates the differences in blood glucose levels after oral treatments with alcoholic extract, saponin, and metformin over 24 hours. The blood glucose level significantly increased in the diabetic group (444.08 ± 10.97, P<0.001) compared to other groups. Treatment with plant extract (P<0.001) was superior to the saponin-treated group (P<0.01) and metformin (P<0.01) group in lowering the blood glucose level.

Plants are valuable resources for drugs, especially in developing nations, where a substantial portion of the world’s population still relies on traditional medicines to treat critical conditions [37, 38]. For diabetes treatment, herbal medicines have been used for a long time and are accepted as a substitute therapy. The active principles found in medicinal plants can repair pancreatic beta cells, combat insulin resistance, or release insulin [39]. In addition, due to their composition of a multitude of chemicals that aid in fighting infection and speeding up wound healing, phytochemicals, and naturally derived substances have gained great advantages over synthetic chemicals [40].

*Phoenix dactylifera* is a member of the genus *Phoenix*, belonging to the family *Arecaceae*. The plant is commonly grown for its edible fruits and kernels. In this work, we studied the effect of the *Phoenix dactylifera* leaf extract on blood glucose levels and wound healing in diabetic and normal rat model. It was reported that the antidiabetic activity of *P*. *dactylifera* extracts due to its saponins, phenol, steroids, and flavonoids contents [17]. Al Dawah & Ibrahim reported that the crude extract of *P*. *dactylifera L* leaves have tannins, steroids, flavonoids, saponins, alkaloids, phenols, and amino acids. Saponins are glycosides promote antioxidants and anti-inflammatory reactions among their other functions [41]. Isolation of triterpene (oleanolic acid), phenolic (vanillyl alcohol), and sterol–glycoside (β sitosterol 3 O β d glucoside) from Saudi date palm leaves were reported [11]. Suleiman et al. reported that palm leaves contain oleanolic acid, vanillyl alcohol, and β sitosterol 3 *O* β d glucoside [42]. Oleanolic acid has been used as a therapeutic agent to improve insulin action, inhibit gluconeogenesis, and promote glucose utilization in diabetic models. This finding may explain the reduction in blood glucose levels upon treatment with leaf extract in the current study.

### Effect on wound healing

#### Incision wound

**Fig 4:**
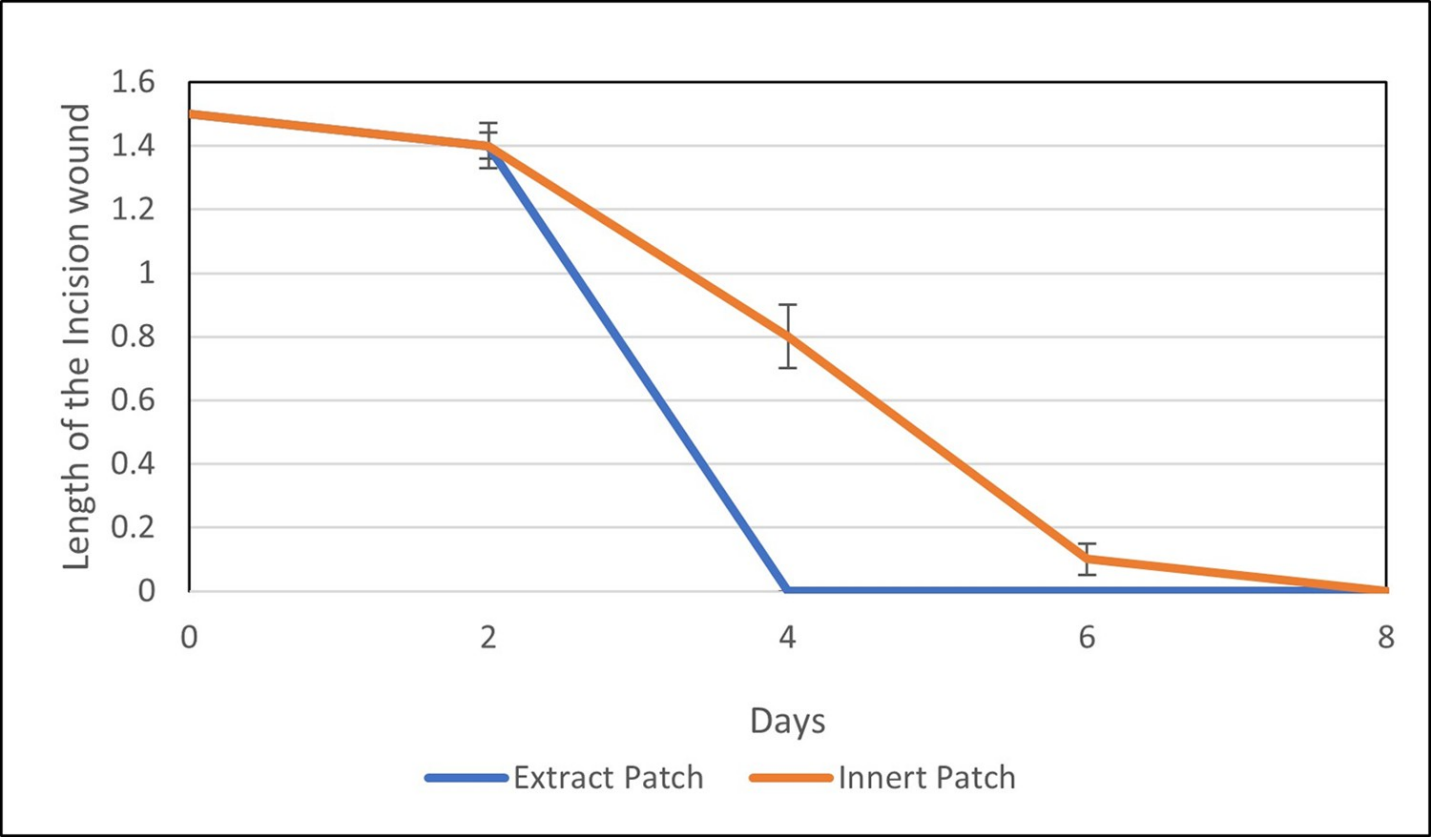
Length of the incision wound (cm) following treatment with extract or inert films.

Fig 4 illustrates the difference between applying the extract and the inert films in incision wound healing time in diabetic rats. Wounds treated with extract healed faster than inert film. There were no significant differences between the healing rates of both groups in the first two days, where the incision wound size decreased from 1.5 cm to 1.4 cm upon applying both films. However, the group that received the extract films showed significantly higher (p <0.05) healing rates with reduced wound size on the following days of the experiment than other groups. On day 4, the incision wound treated with the extract was 100% closed, while the group treated with an inert film was healed on the ninth day.

#### Excision wound healing

**Fig 5:**
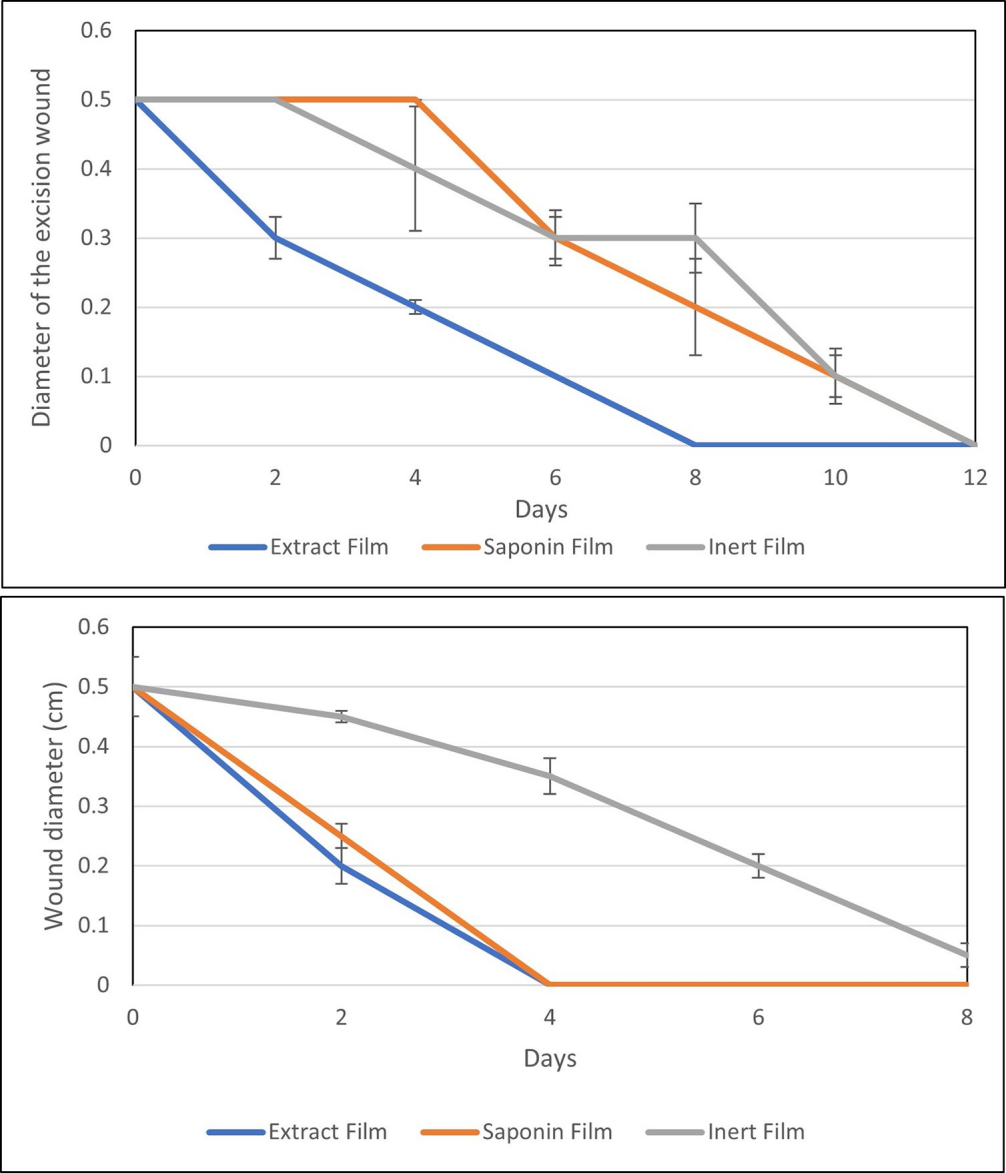
The excision wound’s (cm) diameter after treatment with extract films, saponin films, or inert films in **A**) diabetic and **B**) normal rat models.

Fig 5 illustrates the difference between the application of the extract film, the inert film, and saponin film in excision wound healing time in normal and diabetic rat models. In diabetic rats, the wounds treated with the extract film showed a significant decline in wound size compared to other groups. On the fourth day, the diameter decreased from 0.5 cm to 0.3 cm, whereas the size of the wounds that received an inert and saponin film remained steady. Furthermore, the wounds treated with an extract film were closed on the eighth day. On the other side, the diameter of the wounds that received a saponin film decreased gradually and reached 0.2 cm on the 8^th^ day, decreased by 50% on the 10^th^ day, and then 100% closed on the 12^th^ day. The repeated measures ANOVA confirms the significant difference between the extract-treated wounds and other treatments from the 4^th^ day onwards.

In normal rats with excision wounds, the extract-treated wounds achieved nearly 60% closure of the wound on the 2^nd^ day compared with the 50% wound closure observed in the saponin group and 10% of the control group receiving an inert film. By day four, wounds treated with extract and saponin films were completely closed (100%), while the control wounds remained unhealed even after eight days, demonstrating that treatment accelerated wound healing.

### Histopathological investigations

#### H&E staining

**Fig 6:**
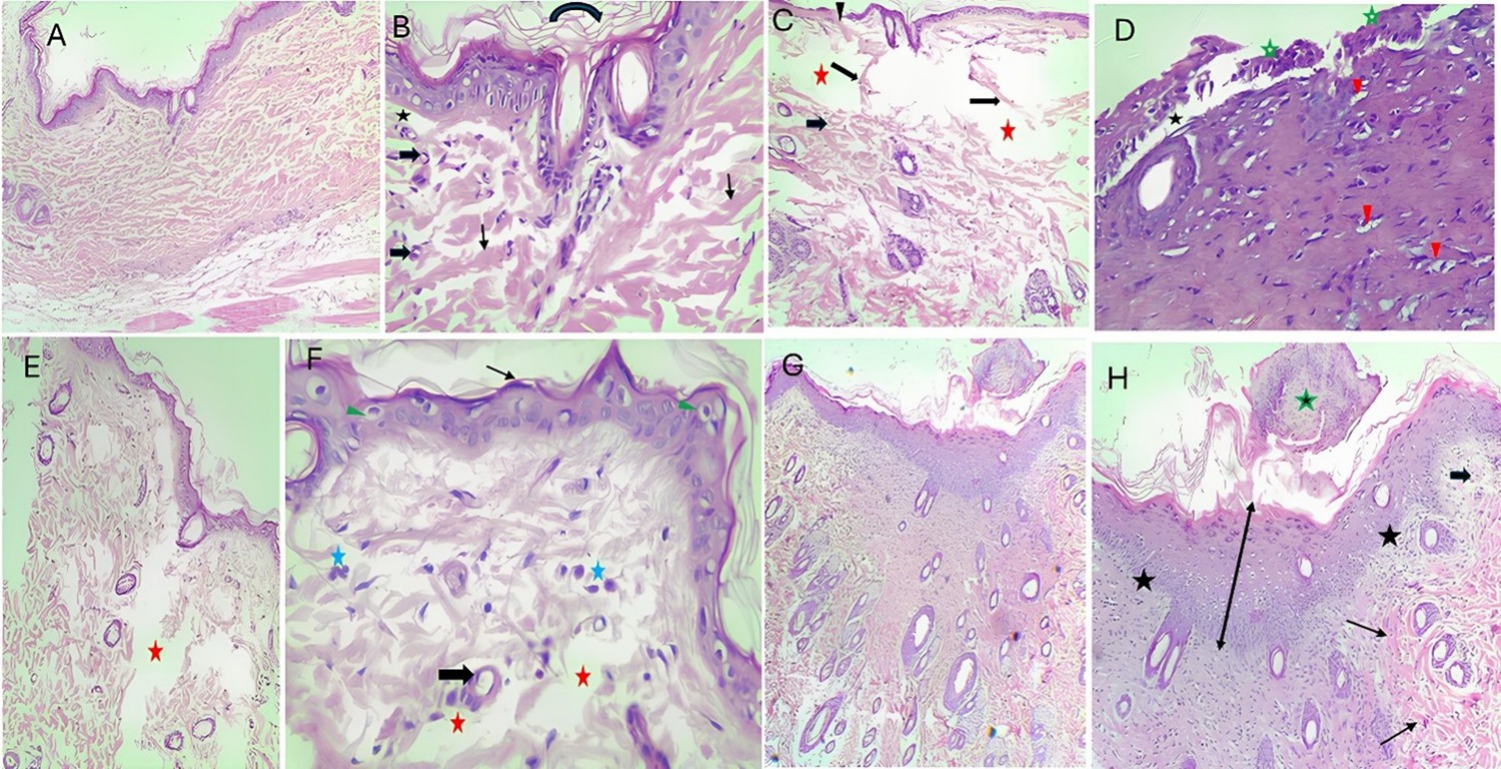
Haematoxylin and eosin-stained sections. **A and B**: Normal control rat healed wound shows complete re-epithelization of the epidermis and full differentiation of keratinocytes with the appearance of a horny layer. Collagen fibers are organized (thin arrow). Some blood vessels (thick arrows) (X100 and X400, respectively). **C and D**: A healed wound of a diabetic rat with inert film reveals incomplete filling of the wound space by granulation tissue (red asterisk) with disorganized collagen fibres (thin arrow). The epidermis is very thin, with areas of incomplete re-epithelization (arrowhead). The dermo-epidermal junction (black asterisk) and some laminated blood vessels are separated. (X100 and X400, respectively). **E and F**: The healed wound of a diabetic rat treated with a saponin film reveals thin, irregular epithelization with incomplete keratinocytes’ differentiation. The superficial cell layer of the epidermis shows flat nuclei. (thin arrow). Inflammatory cells (asterisk) and blood vessels (thick arrow) are visible. (X100 and X400, respectively). **G and H:** The healed wound of a diabetic rat treated with extract film shows full epithelization with complete differentiation of keratinocytes with areas of epithelial hyperplasia. The intact dermo-epidermal junction (thin arrow), invasion of the scab with inflammatory cells (asterisk), and some blood vessels are seen (X100 and X400, respectively).

Complete epithelization of the wound and the presence of a keratinized layer (horny layer) on the surface was observed in the control group. Intact dermo-epidermal junction and reorganization of the dermal collagen fibres with the formation of skin appendages were also observed (Fig 6A and 6B).

However, the cutaneous wounds in non-treated diabetic rats and diabetic rats treated with an inert patch failed to re-epithelize and separated at the dermo-epidermal junction. The dermis revealed more cellular infiltration, disorganized collagen fibres, and incomplete filling of the large wound spaces by granulation tissue. Many laminated blood vessels were observed (Fig 6C and 6D).

The application of saponin film on the wounds of diabetic rats slowly enhanced the process of wound healing to some extent, as confirmed by the epithelization of the epidermis. However, the epidermis appeared thin and irregular, and the dermis showed moderate inflammatory cell infiltration with small areas not filled with granulation tissue (Fig 6E and 6F).

Applying an extract film to the wound of diabetic rats accelerated the wound healing process. This was proved by complete re-epithelization of the epidermis and full keratinocyte differentiation over the whole wound area with areas of epithelial hyperplasia. The dermo-epidermal junction was intact, and the dermis was well-reorganized (Fig 6G and 6H).

Table 6 compares the collagen amount, inflammatory cells, and formation of new capillaries in the normal control group and the diabetic group treated with inert film, saponin film, and extract film, respectively.

**Table 6:** Comparison of the collagen amount, inflammatory cells, and formation of new capillaries in the normal and diabetic group treated with inert, saponin, and extract film.

Item	Normal control	Diabetic rats treated with inert film	Diabetic rats treated with saponin film	Diabetic rats treated with extract film
**Collagen amount**	Normal & organized	Mild & disorganized	Mild-moderate & disorganized	Moderate & organized in most areas
**Inflammatory cells**	Normal	More	Moderate	Mild
**New capillaries**	Normal	More	Few	Normal

### Mallory’s trichrome staining

**Fig 7:**
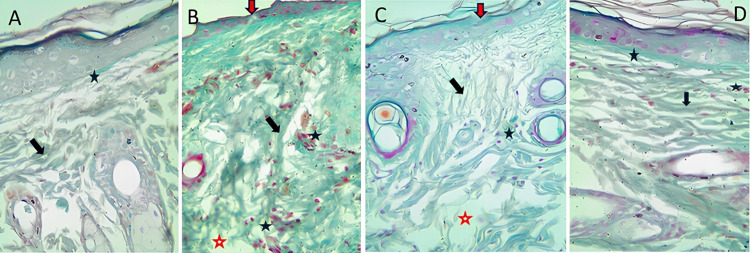
Mallory’s trichrome staining (X400). **A.** A control rat-healed wound reveals an intact dermo-epidermal junction (thin arrow) and organized collagen bundles (thick arrow). **B.** An inert film-treated diabetic rat wound shows disorganized collagen bundles (thick arrow), inflammatory cells (black asterisk), and incomplete filling of the wound space by granulation tissue (red asterisk). **C.** A diabetic rat treated with saponin film reveals disorganized fine immature collagen bundles (thick arrow), inflammatory cells (black asterisk), and incomplete filling of the wound space by granulation tissue (red asterisk). **D.** diabetic rat treated with extract film showing an intact dermo-epidermal junction (thin arrow), organized collagen bundles (thick arrow), and inflammatory cells (asterisk).

Compact collagen bundles were found in the dermis of the normal control group (Fig 7A), while the skin wound of non-treated diabetic rats and diabetic rats treated with an inert film showed randomly scattered collagen deposition (Fig 7B). Applying saponin film on the wound of diabetic rats revealed the deposition of immature collagen fibres in the upper part of the wound. In contrast, the deep part of the wound revealed collagen bundles. Both types of collagens were randomly arranged (Fig 7C). The application of extract film on the diabetic rat wound showed more properly organized collagen bundle deposition (Fig 7D).

Wounds constitute a significant health issue worldwide. The wound healing process is complex and dynamic, entailing the return of damaged tissue layers and cellular structures to their original states. This process includes contracture, epithelization, and connective tissue deposition. During the healing process, the wound area shrinks due to wound contracture, which starts during the fibroblastic stage. The controlled production, deposition, and maturation of new collagen are critical to healing.

Approximately 25% of diabetic patients suffer from impaired wound healing [43]. Treating diabetic wounds has been one of the most challenging clinical problems. Inflammation epithelialization, granulation, neovascularization, and wound contraction are complicated and dynamic wound-healing components [43]. Augmented oxidative stress and reactive oxygen species (ROS) are the most common causes of delayed wound healing in diabetic patients [44]. This raises the risk of infection in the injured area, leading to complicated health issues [45] Therefore, reducing or inhibiting inflammation or free radicals’ formation by presenting antioxidants and anti-inflammatory agents in the treatment process can improve the healing process of diabetic wounds [43].

Both incision and excision wound models were used. The incision wound disappeared on the 4^th^ day when treated with the plant extract film. However, the animals treated with the inert film showed wound closure by the 8^th^ day of the experiment. Masson Meyers et al. demonstrated that the incision wound disappeared on the 14^th^ day without treatment [46]. Upon applying the film with the leaf extract on the excision, wounds were completely closed on the 8^th^ day in the diabetic rats treated with leaf extract film. On the other hand, the wound closed on the 12^th^ day when treated with a saponin film and an inert film. In the normal rats treated with the extract film and saponin film, 100% wound closure was observed on the 6^th^ day, while the wound treated with the inert film was partially closed even after the 8^th^ day.

Palm leaf extract indicated effective wound healing capacity as evident from better wound closure (P < 0.05) compared to saponin and inert film-treated groups using repeated measures ANOVA. A significant difference was observed from day two of the treatment with palm extract compared to the inert patch. It was confirmed by the improvement of tissue regeneration upon histopathological investigations. This could be attributed to the presence of phenolic acids and flavonoids in plant extract. Tannins are phenolic compounds known to have a role in treating inflamed or ulcerated tissues [14]. Flavonoids have previously been reported to be used in wound healing. A phytochemical investigation of the *Phoenix dactylifera* leaf extract was carried out. RP HPLC evaluation of the leaf extracts enabled the identification and quantitation of various flavonoid and phenolic compounds. Apigenin, kaempferol, and rutin were the major flavonoid components, while caffeic acid, p coumaric acid, and ellagic acid were the major phenolic acids components in the leaf extract. The phenolic acids compounds decreased the sugar level in diabetic rats [47]. In addition, it showed wound-healing properties [48].

In an earlier study, the diabetic excision wound treated with a bio-polymeric scaffold incorporated with p-Coumaric acid enhances healing by modulating MMP-9 and TGF-β3 expression [49]. p-Coumaric acid improves the healing process by enhancing angiogenesis, tissue regeneration, and balanced collagen turnover. Syringic acid is one of the abundant phenolic compounds present in olives, dates, spices, pumpkins, and grapes used in various diseases, including diabetes. It possesses antioxidant, antimicrobial, anti-inflammatory, and antiendotoxic activities [50]. The syringic acid inhibited the pro-inflammatory response with improved anti-inflammatory response, inhibited the elevated oxidative stress, and decreased the concentrations of matrix metalloproteinases and increased expression of growth factors with significant improvement in collagen deposition, re-epithelialization, and complete skin structure after treatment of diabetic wounds for 14 days [51]. Similar references are also found for Caffeic acid [52], Ferulic acid [53], Ellagic acid [54], suggesting the beneficial effects of these compounds in diabetic wound healing. Similar work is also found for flavonoids detected in our leaf extract, i.e., Naringin [55], Rutin [56], Quercetin [57], Kaempferol [58], Apigenin [59], and Catechin [60, 61]. All these phytochemicals in our plant extract may result in higher wound-healing properties.

Ginseng saponins showed a better wound-healing effect than the control group [62]. The wound healing activity appeared more in the extract of the leaves than the separated saponin itself; this is attributed to the synergistic activity between the phenolic, flavonoid, steroids, and saponin, which fasting the wound healing. Untreated diabetic skin wounds exhibited ulceration, inflammation, defective granulation tissue formation, and a significant decrease in collagen deposition with disorganization after two weeks. Impaired new blood vessel formation in diabetic rats delayed the healing process and caused ulceration [63]. This might be due to the association between diabetes and the reduction of vascular epithelial growth factor (VEGF) [64]. Diabetic wounds are accompanied by inadequate production of extracellular matrix with proteolysis of dermal protein. This was attributed to the upregulation of the activated metalloproteinase. So, the collagen content in diabetic wounds decreases and subsequently retarded the healing process [65]. The application of extract film on diabetic wounds markedly enhanced the healing process as the wound exhibited complete reepithelization and remodelling of granulation tissue. This stage of wound healing is recognized by increasing collagen content and regular organization, as well as a reduction in the inflammatory cell population.

Re-epithelization is a crucial step for wound healing. It includes the proliferation and migration of keratinocytes from the neighbouring epidermis and the surrounding skin appendages [66]. Macroscopic, morphometric, and histological data suggested that the cutaneous wound healing in rats treated with the leaf extract was better and faster than in the control or inert-treated groups. It is possible that the anti-inflammatory and antioxidant activities of the extract may be associated with wound-healing effects and can be used in wound-healing treatment [18].

## Conclusion

Diabetes mellitus poses significant challenges to individuals’ quality of life, increasing the risk of comorbid diseases and impaired wound healing. This article explored the potential effects of extracting bioactive compounds from young palm leaves for wound healing and antidiabetic activity. The findings highlight the marked effect of *Phoenix dactylifera* extract as a supportive or alternative treatment for both hyperglycemia and wounds. Further research and clinical trials are warranted to validate these findings and explore the underlying mechanisms of action.

## Supporting information

S1 File Calibration curve of extract in methanol. (DOCX)

S1 Graphical abstract (TIF)
