# Supplementary material for: The wound healing and hypoglycemic activates of date palm (Phoenix dactylifera) leaf extract and saponins in diabetic and normal rats
Source: PLoS One. 2024 Sep 23;19(9):e0308879. doi: 10.1371/journal.pone.0308879 (PMC11419346; doi:10.1371/journal.pone.0308879)
Supplement: S1 File — (DOCX) [file pone.0308879.s001.docx]

**Calibration curve of extract in methanol**
