# Supplementary figures and images for: The wound healing and hypoglycemic activates of date palm (Phoenix dactylifera) leaf extract and saponins in diabetic and normal rats
Source: PLoS One. 2024 Sep 23;19(9):e0308879. doi: 10.1371/journal.pone.0308879 (PMC11419346; doi:10.1371/journal.pone.0308879)

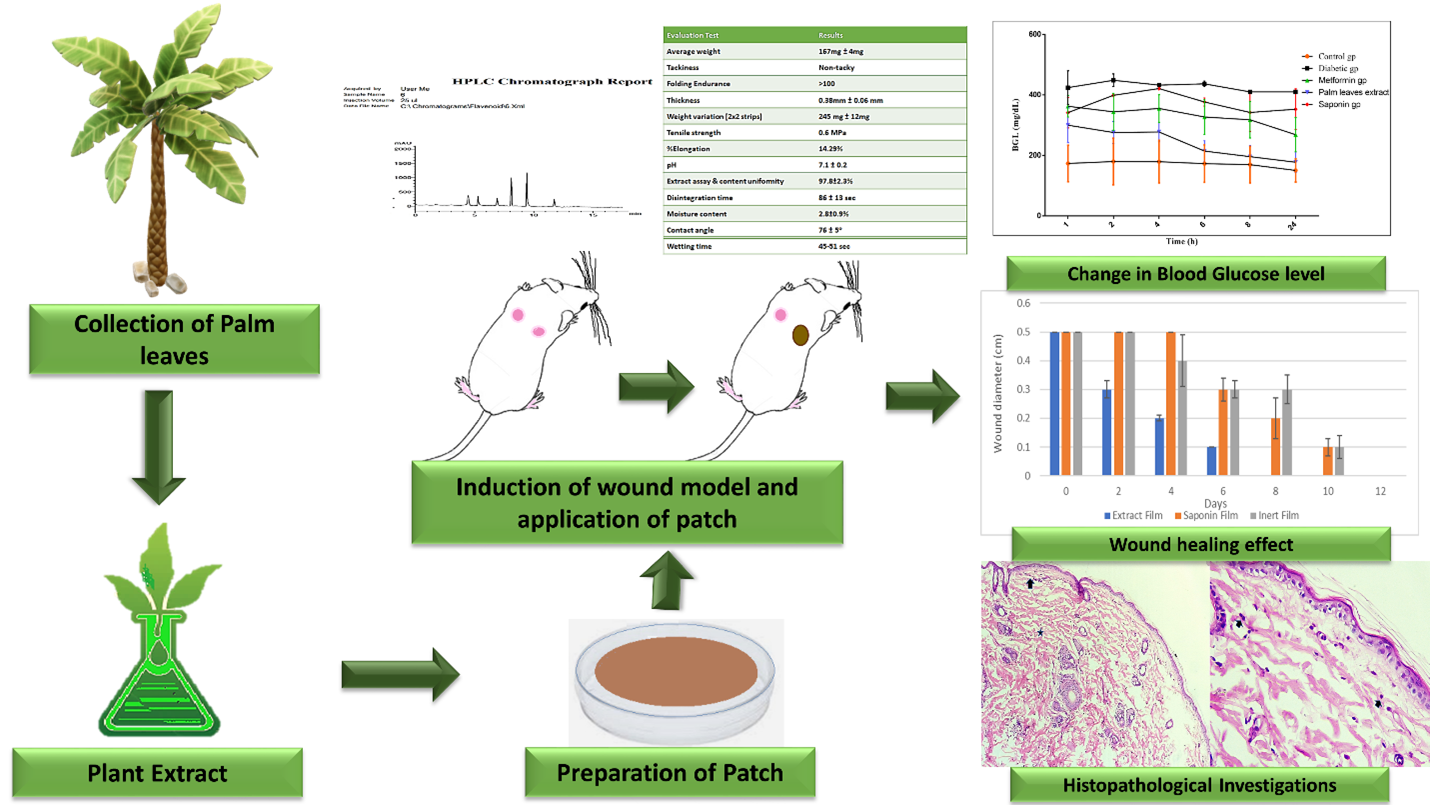

Supplement: S1 Graphical abstract — (TIF) [file pone.0308879.s002.tif]
